# Supplementary figures and images for: Time-Course Transcriptomic Analysis Reveals Molecular Insights into the Inflorescence and Flower Development of Cardiocrinum giganteum
Source: Plants (Basel). 2024 Feb 27;13(5):649. doi: 10.3390/plants13050649 (PMC10934431; doi:10.3390/plants13050649)

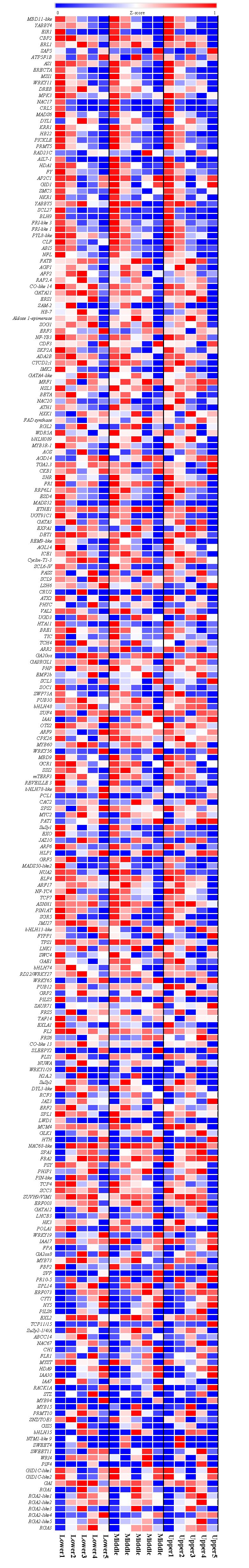

Supplement: Supplementary file 1 [file plants-13-00649-s001.zip › Figure S1.tif]
